# Impact of a 12-Week Core Stability Training on Upper Trunk Stability, Trunk Mobility, and Postural Asymmetries in University Students

**DOI:** 10.3390/life15121801

**Published:** 2025-11-24

**Authors:** Stefan Alecu, Gheorghe Adrian Onea, Dana Badau

**Affiliations:** Faculty of Physical Education and Mountain Sports, Transilvania University, 500068 Brasov, Romania; alecu.stefan@unitbv.ro (S.A.); dana.badau@unitbv.ro (D.B.)

**Keywords:** core stability training, trunk mobility, postural asymmetries, upper trunk stability, Y-Balance Test, university students

## Abstract

The present study aimed to investigate the effects of a 12-week Core Stability Training (CST) program on trunk mobility (twisting and bending), upper quarter dynamic balance (UQ-YBT scores), and postural asymmetry indices (Anterior—ATSI—and Posterior Trunk Symmetry Indices—POTSI) of university students. This study examined the effects of a 12-week CST program on trunk mobility and dynamic balance in 181 university students (70 females, 111 males; aged 18–24 years). Participants completed one supervised 90–100-min session per week. Baseline postural symmetry, assessed with the ATSI and POTSI, showed no sex differences. After training, trunk mobility improved significantly (*p* < 0.001). The female group increased twisting by 5.3° and bending by 6.5 cm, while the male group improved by 4.7–6.1° and 6.0–6.1 cm, respectively (η^2^ = 0.625–0.773). Dynamic balance measured by the UQ-YBT also improved in all directions (*p* < 0.001). Mean gains ranged from 4.0 to 9.7 points, with large effect sizes, especially in males (η^2^ up to 0.842). CST effectively enhanced trunk mobility and upper trunk stability in both sexes. Although postural asymmetry did not change, the program improved neuromuscular control and balance, demonstrating its value in promoting postural health among university students.

## 1. Introduction

Postural asymmetries and trunk dysfunction are frequently observed in university students, often because of sedentary behaviors, prolonged sitting during study, and reduced engagement in structured physical activity [1,2]. Moreover, such habits may promote musculoskeletal imbalances, decrease flexibility, and alter spinal alignment, all of which can impair functional capacity [3]. In fact, deficits in trunk stability and limited mobility are linked with poor posture, reduced motor efficiency, and an increased likelihood of musculoskeletal complaints, such as low back pain and shoulder dysfunction [4,5].

As a result, research in young adults has shown that abnormal sagittal and frontal plane alignment can contribute to reduced physical performance and predispose individuals to overuse injuries [2,6]. Targeted trunk stability interventions have demonstrated positive effects, with evidence showing improvements in push-up performance and overall stability measures in active female students [7,8,9]. Trunk stability, when assessed through proprioceptive neuromuscular facilitation in healthy male university students, has been found to be associated with performance in both upper and lower limb tasks [10]. Emerging evidence suggests that enhanced trunk stability, particularly when facilitated through proprioceptive neuromuscular techniques such as rhythmic stabilization, can significantly improve closed kinetic chain performance in both upper and lower limbs, indicating a regulatory role of the dominant side in neuromuscular coordination and motor output during functional tasks [11,12].

Curriculum-based interventions in female students have also demonstrated improvements in postural stability [13]. Furthermore, cross-sectional studies show considerable prevalence of postural deficits among university students, often correlated with body composition and physical activity levels [14]. Finally, asymmetries in balance and postural stability parameters in young adults are increasingly documented [15].

Upper trunk stability is especially important, as it contributes to both proximal control and distal efficiency. Indeed, efficient stabilization of the thoracic and lumbar spine provides a stable base for limb movement, allowing force to be transferred effectively between the lower and upper extremities [16,17]. In this study, upper trunk stability refers to the neuromuscular control of the thoracic spine and shoulder girdle during movement tasks, with a particular emphasis on segmental alignment, proprioceptive regulation, and resistance to perturbations in static and dynamic postures.

In activities of daily living, such as carrying loads, sitting upright during study, or performing overhead reaching tasks, deficits in upper trunk stability can result in compensatory patterns, fatigue, and eventual musculoskeletal strain [18]. On the other hand, from a sports and exercise perspective, poor trunk stability has been associated with impaired agility, reduced power output, and increased susceptibility to injuries affecting the spine, shoulder, and hip complexes [19,20].

Trunk mobility also plays a vital role in maintaining musculoskeletal health. For instance, adequate thoracic mobility facilitates efficient respiratory mechanics, reduces compensatory lumbar motion, and contributes to global spinal alignment [3,21].

However, restrictions on trunk mobility can lead to increased stiffness, reduced exercise tolerance, and chronic overloading of surrounding tissues. In the student population, reduced trunk mobility is often exacerbated by prolonged sitting, carrying heavy backpacks, and limited engagement in flexibility or movement-based training [3,22]. Consequently, these factors may collectively contribute to postural asymmetries and long-term dysfunction if not addressed early [17,23]. Despite substantial evidence supporting CST for individual outcomes such as strength or balance, no prior study has examined its combined impact on upper trunk stability, trunk mobility, and postural asymmetry in non-athletic university students—a group at high risk due to sedentary behavior.

Postural asymmetries, such as uneven shoulder or pelvic alignment, are common in young adults and may arise from a combination of lifestyle factors, muscle imbalances, and habitual postures [1,2]. Although minor asymmetries may not always cause immediate symptoms, accumulating evidence suggests they are associated with inefficiencies in movement, decreased endurance, and elevated risk of injury when combined with reduced trunk control [6,24]. More importantly, asymmetrical measures can impair balance and postural control, both of which are essential for maintaining physical health and preventing falls in later life [16].

Core stability training (CST) has been recommended as an effective intervention to address neuromuscular deficits that contribute to limited trunk mobility and postural asymmetries. The rationale for CST is rooted in its capacity to enhance the activation and coordination of deep trunk stabilizers such as the transverse abdominis, multifidus, and pelvic floor muscles, which play critical roles in segmental spinal stability and interlimb coordination [16,25]. Core stability plays a central role in postural control by enhancing neuromuscular coordination, proprioceptive feedback, and segmental alignment. Recent meta-analyses have shown that core training significantly improves dynamic balance, not necessarily through structural changes, but via improved sensorimotor control and trunk neuromuscular activation [26,27]. This distinction supports the idea that functional improvements may precede or even occur independently of anatomical adaptations [28]. Moreover, these findings are consistent with performance-oriented outcomes reported in recent analyses comparing instability-based and traditional core training approaches [29]. In addition, evidence suggests that regular CST leads to improvements in balance, postural sway control, and trunk endurance in both healthy and symptomatic populations [17,22]. Thus, repeated activation of trunk muscles in conjunction with limb movements promotes symmetrical recruitment patterns, which may reduce postural asymmetries and improve functional posture [3,17]. Importantly, several studies conducted in collegiate and university populations have demonstrated that 6–12 weeks of CST is sufficient to elicit measurable improvements in trunk strength, stability, and postural control [1,17,22].

Therefore, these findings suggest that targeted programs could serve as a promising preventive approach against postural dysfunctions in young adults, who are at a critical stage for establishing lifelong movement habits [3,17]. By addressing trunk stability, mobility, and asymmetry early, CST may not only improve immediate physical performance but also reduce the long-term risk of musculoskeletal pain and injury [6,23]. Despite this, the combined impact of CST on upper trunk stability, trunk mobility, and postural asymmetries in university students has not been thoroughly investigated. Most existing research examines these outcomes in isolation or in athletic populations, leaving a gap in understanding the holistic benefits of CST in general student populations [16,24].

Based on the previous arguments, the present study aimed to investigate the effects of a 12-week CST program on trunk mobility (twisting and bending), upper quarter dynamic balance (UQ-YBT scores) and postural asymmetry indices (ATSI and POTSI) of university students.

We hypothesized that the 12-week CST program would improve trunk stability and mobility, leading to measurable gains in dynamic balance, neuromuscular control, and overall postural health among university students.

## 2. Materials and Methods

### 2.1. Study Design and Settings

The study was designed as a 12-week pretest–posttest repeated-measures design, conducted between March and May 2025 in the physiotherapy laboratory at the Faculty of Physical Education and Mountain Sports, Transylvania University of Brașov. The study was conducted in accordance with the Declaration of Helsinki, and all participants provided written informed consent and participated voluntarily. Ethical approval was granted by the Ethical Board of the Faculty of Physical Education and Mountain Sports of Transylvania University of Brașov (Approval No. 101/26.03.2025).

### 2.2. Participants

Participants were selected from various academic fields (Table 1) between March and May 2025. The inclusion criteria were (1) aged 18–24 years; (2) ability to perform exercise; (3) no musculoskeletal or neurological disorders. Participants were excluded if they (1) had recent injuries or spine surgery; (2) had contraindications to exercise; (3) had heart rate > 100 bpm at rest; (4) had participated in structured core stability training in the prior 3 months.

The sample size estimation was performed using G*Power 3.1.9.7, a validated software for statistical power analysis in behavioral and biomedical research. Based on a two-way mixed-model ANOVA (Group × Time) with a medium effect size (f = 0.25), α = 0.05, and desired power = 0.95, the analysis indicated that 54 participants were required in total. Under these assumptions, the actual power of the test was 95%, confirming that the design was adequately powered to detect medium-sized effects.

### 2.3. Instruments for Upper Trunk Assessment

#### 2.3.1. Postural Evaluation

Postural asymmetries were evaluated using the Advanced Postural Evaluation and Correction System (APECS) mobile application [30], a validated digital tool for posture screening. We focused on two standardized indices: the Anterior Trunk Symmetry Index (ATSI) and the Posterior Trunk Symmetry Index (POTSI). These indices quantify angular deviations from the vertical axis across multiple anatomical landmarks. The APECS system has demonstrated strong reliability in previous studies (ICC = 0.85–0.92), it is considered valid for asymmetry detection in both clinical and non-clinical populations [25,31]. All evaluations followed a standardized photographic protocol to ensure consistency.

The formula used for ATSI is:

ATSI = (FAI_SN_ + FAI_A_ + FAI_T_) + (HDI_S_ + HDI_A_ + HDI_T_)

POTSI analyzes symmetry in the posterior plane and is based on the same six indices, but with visual references on the back of the body: FAI: FAI_C7—at the level of the C7 vertebra, FAI_A—axillary level, FAI_T—waist level and HDI: HDI_S—acromion, HDI_A—axillary, HDI_T—waist. The formula used for POTSI is:

POTSI = (FAI_C7_ + FAI_A_ + FAI_T_) + (HDI_S_ + HDI_A_ + HDI_T_)

#### 2.3.2. Trunk Mobility

Trunk axial rotation (left/right) was measured in standing using the Goniometry Advance mobile application installed on a smartphone fixed to a horizontal 2-m stick. The method has been validated in previous mobility studies, with intraclass correlation coefficients (ICCs) exceeding 0.90 for inter-rater and intra-rater reliability [28]. For lateral bending, a flexible tape measure was used to record fingertip displacement along the side of the leg, a technique previously used in flexibility research with excellent repeatability (ICC > 0.88) [27].

#### 2.3.3. Upper Trunk Stability, Mobility and Postural Control—Upper Quarter Y-Balance Test (UQ-YBT)

The Upper Quarter Y-Balance Test (UQ-YBT) was used to assess dynamic postural control. This test involves maintaining a single-arm support in a push-up position while reaching in three directions (medial, inferolateral, superolateral) with the contralateral arm. It has been validated as a reliable measure of shoulder and trunk stability (ICC = 0.80–0.91) and shows strong construct validity for neuromuscular performance [32,33,34].

### 2.4. Core Stability Training (CST) Program

The intervention consisted of a 12-week CST program, delivered one time per week under the supervision of the authors of the study as physical education experts. Each session lasted 90–100 min and followed a standardized structure: warm-up (15–20 min), main training phase (55–65 min), and cool-down (10–15 min). The 12-week CST program was divided into three progressive phases: Adaptation & Mobilization (Weeks 1–4), Strength & Flexibility (Weeks 5–8), and Endurance & Integration (Weeks 9–12). Each phase included static and dynamic exercises targeting the core musculature. Exercises included front and side planks, dead bugs, bird dogs, bridge variations, and trunk rotations, progressing toward unstable surfaces and multi-planar patterns. Progression involved both exercise complexity and contraction duration. The 12-week experimental CST program had identical content for both the female and male groups regarding the type of exercises and execution time. The workload was measured by duration rather than by the number of repetitions. A visual overview of the CST progression is provided in Table 2 to summarize the 3-phase logic, weekly structure, and training intensity.

The warm-up included 5 min of low-intensity aerobic activity (jogging or stationary cycling, Rating of Perceived Exertion (RPE) 3–4 on Borg CR-10) followed by dynamic mobility and activation drills (thoracic rotations, walking lunges, pelvic tilts, glute bridges).

The main phase was organized into three progressive 4-week stages, with systematic increases in duration, resistance, complexity, and volume.

- Weeks 1–4 (Adaptation and Mobilization): exercises: Front plank, side plank, bird-dog, dead bug, bridge; prescription: 3 sets of 20–30 s holds or 10–12 repetitions per exercise; intensity: RPE 4–5, “moderate to somewhat hard.”; goal: neuromuscular activation, trunk alignment, static stabilization.
- Weeks 5–8 (Strength and Flexibility Development): exercises: Plank with arm/leg lift, Swiss ball rollouts, resisted trunk rotations with elastic bands, dynamic bird-dog; prescription: 3–4 sets of 30–45 s holds or 12–14 repetitions; intensity: RPE 5–6, “somewhat hard.”; goal: enhance trunk muscle strength, thoracic/hip flexibility, and integration of trunk control with limb movement.
- Weeks 9–12 (Endurance and Integration): exercises: Dynamic plank walkouts, rotational lifts, medicine ball throws (2–6 kg), single-leg bridge with arm reach; prescription: 3–4 sets of 45–60 s holds or 15–18 repetitions; intensity: RPE 6–7, “hard.”; goal: improve endurance, dynamic stabilization, and functional transfer to daily activities.

Cool-down focuing on: static stretching for trunk extensors, hip flexors, and hamstrings, along with controlled breathing exercises.

Load Progression and Monitoring

- Time under tension: increased progressively (20–30 s → 45–60 s).
- Repetitions/sets: increased gradually (10–12 → 15–18 reps; 3 → 4 sets).
- Resistance: progressed via elastic bands (light → medium → heavy tension) and medicine ball load (2 → 6 kg).
- Complexity: progressed from static → dynamic → unstable and multiplanar tasks.
- Intensity monitoring: Borg CR-10 scale; target RPE increased across phases (4–5 → 6–7).

### 2.5. Statistical Analysis

All data were analyzed using IBM SPSS Statistics v27.0 (IBM Corp., Armonk, NY, USA). Data were first checked for normality of distribution using the Shapiro–Wilk test and for homogeneity of variances using Levene’s test. Only variables meeting these assumptions were analyzed with parametric tests. To examine differences (rather than patterns or associations) in outcomes between groups and across time, a 2 × 2 mixed-model ANOVA was applied, with factors Group (male vs. female) and Time (pre-test vs. post-test). Where repeated measures violated sphericity assumptions, corrections were applied using the Greenhouse–Geisser adjustment. Post hoc pairwise comparisons were adjusted using the Bonferroni correction to control familywise error rates in multiple testing. Effect sizes were reported as partial eta squared (η^2^p). The criteria for interpreting effect sizes were small = 0.01, medium = 0.06, and large = 0.14. Results are expressed as mean ± SD, mean differences (Δ), F-values, *p*-values, and η^2^p. For between-group baseline comparisons, independent-samples *t*-tests were conducted. Statistical significance was set at *p* < 0.05 for all tests. All variables met the assumption of normality (*p* > 0.05) and homogeneity of variances. Sphericity was assessed with Mauchly’s test, and when violated, the Greenhouse–Geisser correction was applied.

## 3. Results

### 3.1. Effects of CST Intervention

#### Participant Anthropometric and Trunk Characteristics

The descriptive statistics for the study population are presented in Table 3. The female group (*n* = 70) had a mean age of 20.18 ± 2.32 years, mean height of 164.87 ± 5.66 cm, and mean weight of 60.34 ± 11.08 kg. The male group (*n* = 111) had a mean age of 19.89 ± 1.55 years, mean height of 176.52 ± 7.59 cm, and mean weight of 74.56 ± 14.77 kg.

Between-group comparisons using one-way ANOVA revealed statistically significant sex differences for height (F = 121.89, *p* < 0.001) and weight (F = 47.88, *p* < 0.001). No significant differences were observed for age (F = 1.04, *p* = 0.309), Anterior Trunk Symmetry Index (ATSI) (F = 1.25, *p* = 0.265), or Posterior Trunk Symmetry Index (POTSI) (F = 0.90, *p* = 0.344).

These findings indicate that, while expected anthropometric differences were present between males and females in terms of height and body weight, trunk symmetry indices did not differ significantly. This suggests that the participants included in the study exhibited comparable postural development across sexes.

### 3.2. Sex-Based Comparisons

The analysis of trunk elasticity tests demonstrated significant improvements from baseline to post-intervention across all conditions, with consistent response patterns that were not influenced by sex (Table 4).

No significant sex × time interactions were identified for either twisting or bending (all F < 2.0, *p* > 0.05), indicating that the relative magnitude of improvements was comparable between males and females. Collectively, these findings confirm consistent training adaptations in trunk elasticity, with bending movements eliciting greater absolute gains than twisting movements, and no evidence of sex-specific differences in responsiveness to the CST intervention.

The between-group analysis (female vs. male participants) for trunk mobility is presented in Table 5 and showed no significant differences for trunk twisting between sexes at the initial assessment (left: F = 0.085, *p* = 0.770; right: F = 0.031, *p* = 0.861) or at the final assessment (left: F = 0.017, *p* = 0.897; right: F = 0.016, *p* = 0.900). These findings suggest that rotational trunk mobility was comparable between males and females both before and after the intervention.

Table 6 presents the analysis of Upper Quarter Y-Balance Test (UQYBT), which revealed significant improvements from the initial test (IT) to the final test (FT) across all conditions, with notable gender-specific response patterns. The most notable finding was the contrasting pattern in upper limb right Direction A, where males showed exceptional improvement (mean difference: 9.716, η^2^ = 0.842) compared to females (mean difference: 6.320, η^2^ = 0.674). Males demonstrated superior absolute performance in directions B and C (ranging from 61.858–80.264) compared to females (47.564–62.464). At the same time, Direction A showed mixed patterns with females achieving comparable or superior performance in the left limb.

In Table 7, the analysis of upper limb Y-test performance revealed highly significant gender differences across all comparisons (*p* < 0.001), with F-values ranging from 12.486 to 69.809. Initial testing demonstrated stronger discrimination between genders compared to final testing, with the left upper limb showing consistently higher F-values than the right limb across both conditions.

The analysis of effect sizes in Table 8 revealed important distinctions in how different trunk and balance measures responded to the 12-week core stability training program. Overall, trunk bending emerged as the most responsive functional domain, while rotational mobility exhibited only modest gains. The consistency of results across genders suggests that male and female participants responded similarly to the intervention, with no meaningful gender-based differences in effect size magnitude.

Effect size estimates (Cohen’s *d*) were computed for pre-post changes. All trunk bending effects were large, (d > 0.5) and very large (d > 0.8), while twisting and balance gains ranged from small to medium.

## 4. Discussion

### 4.1. Postural Asymmetries (ATSI, POTSI)

ATSI and POTSI were used only at baseline to assess the participants’ initial postural alignment and quantify existing asymmetries [15]. These measures provided an overview of structural posture but were not repeated after the intervention because the main objective of the study was to evaluate functional rather than morphological outcomes [21]. Structural asymmetries typically require longer training durations or specific corrective approaches to produce measurable changes, as short-term CST interventions mainly target neuromuscular rather than skeletal adaptations [22]. Previous studies support this interpretation, showing minimal alterations in postural symmetry indices after core training lasting less than three months [15,21]. It has also been demonstrated that structural realignment requires multimodal interventions combining CST with stretching, posture correction, and flexibility training. Our data, therefore, support the hypothesis that functional gains in trunk control and balance can occur independently of static structural adaptations, emphasizing the neuromuscular rather than anatomical basis of early CST responses [22].

### 4.2. Trunk Mobility

The analysis revealed significant pre–post improvements in both trunk twisting and lateral bending, with the most notable increases observed in bending movements. Trunk twisting increased by 4.7–5.3° (*p* < 0.001, η^2^ = 0.64–0.74), while trunk bending improved by 6.1–6.5 cm (*p* < 0.001, η^2^ = 0.67–0.77), indicating large effects. Dynamic stability, assessed through the Y-Balance Test, also showed meaningful gains of 4.0–9.7 points (*p* < 0.001, η^2^ = 0.11–0.84), reflecting improved upper trunk and shoulder control. These changes reflect enhanced flexibility and coordination of the trunk muscles following CST and confirm the program’s effectiveness in promoting functional mobility [3]. The improvement in lateral bending suggests better control of the oblique and paraspinal muscles, which play a key role in segmental stabilization and movement efficiency [5]. Similar trends have been documented in previous trials where participants exhibited measurable gains in spinal range of motion after 6–8 weeks of structured core training, demonstrating the adaptability of the neuromuscular system to repeated stabilization exercises [3,5]. Core stabilization exercises have also been shown to improve horizontal trunk rotation and thoracic flexibility, likely due to increased recruitment of deep stabilizers such as the multifidus and transverse abdominis. Our findings confirm that even in non-athletic university students, progressive CST enhances mobility through the combined effects of greater neuromuscular activation, proprioceptive refinement, and improved intersegmental coordination [17].

### 4.3. Upper Trunk Stability and Dynamic Balance (UQ-YBT)

Participants demonstrated consistent and statistically significant progress in all Y-Balance Test directions following CST, showing marked improvements in medial, inferolateral, and superolateral reaches. These results indicate improved shoulder girdle and upper trunk stability, which are essential for maintaining postural control in closed kinetic chain tasks [16]. This trend aligns with earlier evidence indicating that structured core training enhances neuromuscular coordination and proximal stability, resulting in improved dynamic postural performance [17]. Both reported moderate to large Y-Balance Test gains following core-focused interventions in student and youth groups, reinforcing the positive transfer of CST to balance-related functions [17,20]. Likewise, stability-oriented programs improve trunk control and movement precision during dynamic activities, confirming that core strength directly contributes to better limb coordination [26,35]. These outcomes are explained by improved synchronization of deep stabilizers such as the transverse abdominis, multifidus, and internal obliques, which provide segmental stiffness and optimize movement transfer between the upper and lower body [17]. Our findings reinforce that dynamic stability adapts rapidly to systematic CST, demonstrating that neuromuscular improvements can be achieved within a 12-week intervention [20]. Beyond the functional gains observed in mobility and dynamic balance, the improvements can be attributed to physiological mechanisms underlying core stability training. Notably, CST promotes intersegmental control by enhancing segmental dissociation and stabilizer recruitment patterns within the spine. This neuromuscular refinement allows the thoracolumbar junction to maintain alignment under load, reducing energy leaks during movement. Furthermore, CST enhances intramuscular coordination, particularly within deep trunk muscles such as the transverse abdominis and multifidus, optimizing the timing and force of contraction. The synergy between local stabilizers and global movers also contributes to improved trunk stiffness and dynamic balance, explaining the enhanced Y-Balance performance. These adaptations reflect neuromuscular plasticity and proprioceptive recalibration, a hallmark of effective sensorimotor training.

### 4.4. Exploratory Gender Comparisons

When comparing responses between male and female participants, both groups displayed similar improvement patterns across all main variables, confirming that CST benefits were not sex dependent [36]. These findings align with previous studies that observed comparable Y-Balance and stability gains between men and women when the exercise structure and supervision level were standardized [37]. Although males generally demonstrated slightly higher absolute reach distances, females exhibited greater consistency across test directions, a trend that has been attributed to differences in limb length, motor control strategies, and muscle endurance [38]. These small variations are not indicative of unequal training effectiveness but rather reflect physiological and biomechanical diversity [39]. The overall pattern supports the notion that CST elicits similar neuromuscular adaptations in both sexes when training volume and progression are equivalent [40].

While the current findings align with prior research demonstrating improvements in trunk mobility and balance through CST, it is important to recognize that not all studies report such consistent outcomes. For instance, some trials have shown dynamic stability following short-term interventions, particularly in populations with pre-existing motor control deficits or high baseline performance. Differences in methodology, duration, and training load likely contribute to such discrepancies. Our results may reflect the structured progression and supervision applied throughout the 12-week program. In this context, the study by Thomas E et al. provides valuable insight. It highlights the importance of integrating neuromuscular activation patterns and postural sway analysis to better understand control mechanisms in both stable and unstable positions. Future core stability interventions may benefit from incorporating such multimodal assessments to more precisely quantify intersegmental and proprioceptive improvements [41].

### 4.5. Practical Implications

A 12-week supervised CST program effectively improved trunk mobility, upper trunk stability, and dynamic balance in university students [3,16]. These results reinforce previous evidence identifying CST as a practical and efficient method to mitigate the negative postural effects of sedentary behavior and prolonged sitting [33]. The absence of measurable changes in static postural asymmetry suggests that short-term CST primarily drives neuromuscular rather than morphological adaptations [22]. To achieve lasting structural realignment, longer or multimodal interventions integrating corrective, stretching, and flexibility components are recommended [1,22]. Overall, CST represents an evidence-based, accessible, and low-cost strategy to enhance trunk function, postural control, and movement quality in young adults, making it a valuable addition to university physical education and health promotion programs [1,3,42,43].

### 4.6. Limitations and Future Directions

Despite the promising outcomes, several limitations should be acknowledged. First, although the sample was sufficiently powered to detect medium effect sizes, it consisted exclusively of untrained university students aged 18–24. As such, the results cannot be generalized to athletic, clinical, or older adult populations. Second, the intervention spanned only 12 weeks, which may be insufficient to induce structural changes in postural asymmetry, particularly when targeting deeply rooted musculoskeletal imbalances.

The programs were designed to be identical in duration. The absence of precise monitoring of the number of repetitions for the two groups by gender (the exercises were structured based on time rather than repetitions), as well as the lack of monitoring of effort intensity (heart rate and respiratory rate), can be considered additional limitations of the study. In addition, no post-study assessment was conducted, which hinders understanding of the retention or durability of neuromuscular gains over time.

Future research should explore the minimum effective duration for observable changes in asymmetry, the dose–response relationship of CST progression, the addition of multimodal interventions (e.g., stretching, manual therapy, sensorimotor training), longer-term outcomes via follow-up measurements at 3–6 months and also the use of advanced posturographic tools or EMG analysis to capture subtle adaptations. Comparative trials in athletic and clinical populations would also help determine whether CST offers differential effects based on baseline function or training history.

## 5. Conclusions

This study demonstrated that a 12-week structured core stability training (CST) program enhanced trunk mobility and upper quarter dynamic balance in university students, with benefits evident in both males and females. Improvements were consistent across twisting, bending, and Y-Balance Test performance, confirming CST as an effective intervention to counteract sedentary-related impairments in young adults.

Taken together, these findings highlight CST as a safe, accessible, and evidence-based strategy for promoting functional health and preventing musculoskeletal risks in higher-education settings. The results also underscore the importance of considering multimodal or longer-term approaches when targeting postural asymmetries. Future studies should explore the durability of these adaptations, the underlying neuromechanical mechanisms, and the potential for CST to reduce injury risk over the long term.

These findings support the value of CST as a preventive and functional tool for improving dynamic postural control, but the conclusions must be interpreted with caution. The results are limited to non-athletic, healthy university students, and cannot be generalized to trained athletes, individuals with neuromuscular disorders, or clinical populations.

## Figures and Tables

**Table 1:** Academic fields distribution of the subjects.

Faculty	N	%
Physical Education and Mountain Sports	40	22.1
Medicine	38	21
Mechanical Engineering	35	19.3
Psychology and Education Sciences	34	18.8
Economic Sciences and Business Administration	34	18.8

**Table 2:** Structure of the 12-Week Core Stability Training (CST) program, outlining the three progressive phases, training focus, sample exercises, and intensity (RPE).

Phase	Weeks	Focus	Example Exercises	RPE
Adaptation & Mobilization	1–4	Neuromuscular activation & alignment	Front plank, side plank, bird-dog, dead bug, bridge	4–5
Strength & Flexibility	5–8	Strength, thoracic/hip flexibility	Plank with arm/leg lift, Swiss ball rollouts, resisted rotations	5–6
Endurance & Integration	9–12	Dynamic stabilization & function	Plank walkouts, medicine ball throws, single-leg bridge with reach	6–7

RPE—Rate of Perceived Exertion.

**Table 3:** Distribution and statistical processing of the characteristics of the study subjects.

Characteristics	Gender	N	Mean	SD	ANOVA			
					Mean Square	F	*p*	η^2^
Age	Female	70	20.185	2.323	3.706	1.041	0.309	0.006
	Male	111	19.891	1.551				
Height	Female	70	164.871	5.666	5827.414	121.893	<0.001 *	0.407
	Male	111	176.522	7.592				
Weight	Female	70	60.344	11.077	8684.445	47.881	<0.001 *	0.210
	Male	111	74.567	14.770				
ATSI	Female	70	19.428	10.526	129.205	1.249	0.265	0.007
	Male	111	17.693	9.943				
POTSI	Female	70	11.971	6.988	34.722	0.901	0.344	0.005
	Male	111	11.072	5.664				

N—Number, F—F ratio, *p*—level of probability, ATSI—Anterior Trunk Symmetry Index, POTSI—Posterior Trunk Symmetry Index. * Statistically significant difference between sexes at *p* < 0.05.

**Table 4:** Descriptive and General Linear Model statistical analysis for the female and male groups for Trunk Mobility Tests.

Trunk Mobility Tests	Direction	Sex	Initial Testing Mean	Final Testing Mean	Bonferroni	Greenhouse-Geisser		
					Dif. Mean (FT-IT) [95% CI]	F	*p*	η^2^
Trunk Twisting (grade)	Left	F	136.73 ± 25.98	142.04 ± 25.53	5.32 * [4.61–6.03]	193.954	<0.001	0.738
		M	137.80 ± 22.76	142.52 ± 22.82	4.71 * [4.10–5.32]	183.353	<0.001	0.625
	Right	F	139.16 ± 27.21	144.35 ± 27.60	5.19 * [4.41–5.97]	150.363	<0.001	0.685
		M	139.82 ± 22.98	144.82 ± 22.76	5.01 * [4.32–5.70]	197.826	<0.001	0.643
Trunk Bending (cm)	Left	F	22.58 ± 5.76	29.10 ± 7.55	6.52 * [5.87–7.17]	183.888	<0.001	0.727
		M	23.71 ± 5.46	29.85 ± 7.35	6.14 * [5.58–6.70]	224.595	<0.001	0.671
	Right	F	21.49 ± 5.51	28.01 ± 6.85	6.52 * [5.88–7.16]	234.300	<0.001	0.773
		M	23.81 ± 5.50	29.87 ± 7.37	6.06 * [5.48–6.64]	220.901	<0.001	0.668

* The mean difference is significant at the 0.05 level. Adjustment for multiple comparisons: Bonferroni; η^2^—partial Eta Squared, IT—initial test, FT—final test, CI—confidence interval.

**Table 5:** ANOVA analysis between female and male groups for Trunk mobility Tests.

Motor Test	Direction	Test	Sum of Squares	Mean Square	F	*p*	η^2^
Trunk Twisting (grade)	Left	Inital test	49.446	49.446	0.085	0.770	0.001
	Right		18.852	18.852	0.031	0.861	0.000
Trunk Bending (cm)	Left		54.740	54.740	1.759	0.186	0.010
	Right		231.398	231.398	7.652	0.006	0.045
Trunk Twisting (grade)	Left	Final test	9.561	9.561	0.017	0.897	0.000
	Right		9.600	9.600	0.016	0.900	0.000
Trunk Bending (cm)	Left		24.084	24.084	0.437	0.510	0.003
	Right		148.617	148.617	2.885	0.091	0.017

**Table 6:** Descriptive and General Linear Model statistical analysis for the female and male groups for Y-Test upper limbs.

Limb	Direction		Test	Mean	SD	Bonferroni	Greenhouse-Geisser		
						Di. Mean (FT-IT)	F	*p*	η^2^
Upper limb left	(A) lateral left	Female	IT	64.954	9.196	6.313 *	193.034	<0.001	0.737
			FT	71.267	10.146				
		Male	IT	64.954	9.196	4.629 *	155.231	<0.001	0.692
			FT	69.582	13.409				
	(B) right posterior oblique	Female	IT	54.711	11.579	6.379 *	195.989	<0.001	0.740
			FT	61.090	12.157				
		Male	IT	75.171	16.542	5.093 *	16.360	<0.001	0.129
			FT	80.264	13.471				
	(C) right anterior oblique	Female	IT	48.301	11.012	6.700 *	170.683	<0.001	0.712
			FT	55.001	11.503				
		Male	IT	61.858	13.418	7.102 *	32.012	<0.001	0.225
			FT	68.960	15.371				
Upper limb right	(A) lateral right	Female	IT	65.480	10.928	6.320 *	142.836	<0.001	0.674
			FT	71.800	11.192				
		Male	IT	55.757	12.447	9.716 *	584.646	<0.001	0.842
			FT	65.473	16.024				
	(B) oblique-posterior left	Female	IT	55.997	12.713	6.467 *	184.586	<0.001	0.728
			FT	62.464	13.391				
		Male	IT	75.181	17.015	3.996 *	14.252	<0.001	0.115
			FT	79.177	14.788				
	(C) oblique-anterior left	Female	IT	47.564	11.789	6.953 *	254.990	<0.001	0.787
			FT	54.517	11.793				
		Male	IT	61.782	13.006	7.611 *	45.891	<0.001	0.294
			FT	69.393	16.005				

* The mean difference is significant at the 0.05 level. Adjustment for multiple comparisons: Bonferroni; IT—initial test, FT—final test.

**Table 7:** ANOVA analysis between female and male groups for Y Test of upper limbs.

Limb	Direction	Test	Sum of Squares	Mean Square	F	*p*
Upper limb left	(A) lateral left	IT	10,061.774	10,061.774	69.809	<0.001
	(B) right posterior oblique		8715.796	8715.796	44.267	<0.001
	(C) right anterior oblique		2386.607	2386.607	16.811	<0.001
Upper limb right	(A) lateral right		8054.223	8054.223	44.636	<0.001
	(B) oblique-posterior left		7704.216	7704.216	35.061	<0.001
	(C) oblique-anterior left		2792.537	2792.537	18.294	<0.001
Upper limb left	(A) left lateral	FT	9406.962	9406.962	58.098	<0.001
	(B) right posterior oblique		8511.772	8511.772	37.806	<0.001
	(C) right anterior oblique		2018.493	2018.493	12.486	<0.001
Upper limb right	(A) lateral right		8026.488	8026.488	38.946	<0.001
	(B) oblique-posterior left		6942.211	6942.211	28.101	<0.001
	(C) oblique-anterior left		2266.220	2266.220	14.382	<0.001

Upper limb left (A) left lateral, (B) right posterior oblique, (C) right anterior oblique. Upper limb right (A) lateral-right (A), (B) oblique-posterior left, (C) oblique-anterior left. IT—initial test, FT—final test.

**Table 8:** Cohen’s *d* effect sizes for key outcomes (male vs. female).

Outcome	Group	Pre Mean ± SD	Post Mean ± SD	*d*
Trunk Twisting (Left)	Male	137.80 ± 22.76	142.52 ± 22.82	0.21
Trunk Twisting (Left)	Female	136.73 ± 25.98	142.04 ± 25.53	0.21
Trunk Bending (Right)	Male	23.81 ± 5.50	29.87 ± 7.37	0.93
Trunk Bending (Right)	Female	21.49 ± 5.51	28.01 ± 6.85	1.05
Y-Balance A (Right Arm)	Male	55.76 ± 12.45	65.47 ± 16.02	0.68
Y-Balance A (Right Arm)	Female	65.48 ± 10.93	71.80 ± 11.19	0.56

*d*—Cohen’s d value.

## Data Availability

The original contributions presented in this study are included in the article; further inquiries can be directed to the first and corresponding author.
